# Analysis of mortality in low-risk patients undergoing coronary artery bypass grafting

**DOI:** 10.5830/CVJA-2013-040

**Published:** 2013-10

**Authors:** Canturk Cakalagaoglu, Cengiz Koksal, Taylan Adademir, Ali Fedakar, Mustafa Yildiz, Müslüm Şahin, Fikri Kutlay, Besim Yigiter

**Affiliations:** Cardiovascular Surgery Clinic, Kartal Kosuyolu Research and Training Hospital, Kartal, Istanbul, Turkey; Cardiovascular Surgery Clinic, Kartal Kosuyolu Research and Training Hospital, Kartal, Istanbul, Turkey; Cardiovascular Surgery Clinic, Kartal Kosuyolu Research and Training Hospital, Kartal, Istanbul, Turkey; Cardiovascular Surgery Clinic, Kartal Kosuyolu Research and Training Hospital, Kartal, Istanbul, Turkey; Department of Cardiology, Sakarya University, Sakarya, Turkey; Cardiology Clinic, Kartal Kosuyolu Research and Training Hospital, Kartal, Istanbul, Turkey; Cardiovascular Surgery Clinic, Veni Vidi Hospital, Dagkapi, Diyarbakir, Turkey; Cardiovascular Surgery Clinic, Isvicre Hospital, Icerenkoy, Istanbul, Turkey

**Keywords:** coronary artery bypass grafting, myocardial infarction, risk assessment, low risk

## Abstract

**Aim:**

The aims of this study were to determine the early mortality rate in low-risk coronary artery bypass graft (CABG) patients and examine the causes of death, to identify problems that could be avoided in future surgeries.

**Methods:**

All low-risk patients (EuroSCORE ≤ 2) who died after CABG were included. Their peri-operative information was meticulously studied by internal and independent external reviewers to identify causes of death, which were classified as: cardiac or non-cardiac; and a further division as: (1) non-preventable, (2) preventable (technical error), and (3) preventable (system error).

**Results:**

Early mortality was 0.93% (24/2 570). Eleven patients (45.8%) were classified as preventable deaths. In six of them the main problem was identified as graft thrombosis, which was secondary to a technical error of either the harvesting or anastomosis of the left internal mammarian artery. There were also five system errors identified as delays in the treatment of an identified and potentially reversible problem.

**Conclusions:**

Correction of technical and system errors, such as harvesting of the left internal mammarian artery, haemostasis during surgery, and establishing standard protocols for the transfer of patients from ward to intensive care units will eventually lead to improvement in both the quality of care and patient outcomes, even in low-risk groups.

## Abstract

Scoring systems that predict the risk of operative mortality have been under development for more than a decade and one of the most frequently used systems, EuroSCORE, has been established.^1^,^2^ EuroSCORE divides patients into three risk groups, based on the score obtained during assessment: low-risk patients (value ≤ 2) have a predicted mortality of 1.27–1.29% and an observed mortality of 0.4–1.0%.^2^-^7^

Although the reasons for mortality have been extensively studied in high-risk patients,^8^,^9^ only a few reports have analysed the reasons for and possible preventive strategies of mortality in low-risk patients undergoing coronary artery bypass grafting (CABG).^10^,^11^ Drawing on the FIASCO study,^10^ we reviewed the mortality in our own low-risk CABG patient population in order to identify whether death could be considered preventable, and if so, whether it was due to a technical or system error.

## Methods

The study was approved by the local hospital ethics committee. All cardiac surgical patients were prospectively risk stratified using the additive and logistic EuroSCORE.^12^ The cardiac surgery unit recorded all patients’ characteristics, operation details and postoperative outcomes contemporaneously in a computer database. Patients with an additive EuroSCORE ≤ 2 who died in the early postoperative period (death from any cause within 30 days of the operation) were identified from the database.

For the study, patient inclusion criteria were: women undergoing CABG with no other risk factors (one point) or with any risk factor adding one point (two points), and men undergoing CABG with one or two risk factors of one point or with one two-point risk factor. We exluded patients with moderate and high EuroSCOREs. All of the operations were performed on-pump; combined operations (CABG + heart vessel/carotid/aorta surgery/etc.) and emergencies surgeries were also excluded.

The details of each case were reviewed and analysed by the cardiac surgery and anaesthetic teams. The details of the patients were also reviewed by an independent surgeon from another hospital, who has considerable experience in assessing patients’ hospital records. The cause of death of each patient was identified and a decision was made to define the death as cardiac or non-cardiac related. Also, as in the FIASCO study, deaths were further classified into three categories: (1) non-preventable, (2) preventable (technical error), and (3) preventable (system error), in order to determine the cause of death.^10^

## Results

Between 2002 and 2007, 3 729 patients underwent on-pump CABG surgery at our hospital and 2 570 (69%) of them were identified as having an additive EuroSCORE ≤ 2. They were categorised as a low-risk group according to the EuroSCORE definition.^2^,^3^ There were 24 early mortalities (defined as occurring within 30 days of the CABG operation) in the study group and therefore mortality was found to be 0.93% (24 out of 2 570). The deaths were further classified as cardiac and non-cardiac, and according to this division, nine (37.5%) of the deaths were found to have cardiac causes, whereas 15 (62.5%) were considered to have a non-cardiac cause.

As in the FIASCO study, when deaths were classified into non-preventable, preventable (technical error) or preventable (system error),^10^ 11 (45.8%) of the deaths were considered to be preventable and 13 (54.2%) were non-preventable, a categorisation which both internal and external reviewers agreed upon. The details of patient deaths are summarised in Table 1. All patients in this group received left internal mammary artery grafts for revascularisation of the left anterior descending artery. Other grafts were obtained from veins, and on average each patient received 2.9 ± 0.7 bypass grafts.

**Table 1:** Summary Of The Cause Of Death And Possible Identified Problems In Low-Risk CABG Operations

*No*	*Age*	*Gender*	*Cause of death*	*Cardiac death*	*Preventable*	*Identified problem*
1	56	Male	Cardiac arrest in the ICU, peri-operative MI	Yes	Yes – technical	Haematoma of LIMA, occlusion of graft
2	64	Male	Cardiac arrest in the ICU, peri-operative MI	Yes	Yes – technical	Dissection of LIMA, occlusion of graft
3	63	Male	Cardiac arrest in the ward, peri-operative MI	Yes	Yes – technical	Haematoma of LIMA, occlusion of graft
4	58	Male	Cardiac arrest in the ICU, peri-operative MI	Yes	Yes – technical	Dissection of LIMA, occlusion of graft
5	59	Female	Cardiac arrest in the ICU, peri-operative MI	Yes	Yes – technical	Haematoma of LIMA, occlusion of graft
6	54	Male	Ventricular fibrillation after CABG × 3 in ICU, 2 saphenous vein occlusions	Yes	Yes – technical	Surgical technical error of anastomosis?
7	57	Female	Renal failure (excessive blood transfusion) 2 800 mm drainage, late revision surgery	No	Yes – system	System error
8	61	Male	Renal failure ( excessive blood transfusion) 3 650 mm^3^ drainage, no revision	No	Yes – system	System error
9	53	Male	Pneumothorax-related respiratory insufficiency in ward	No	Yes – system	System error, unable to transfer to ICU on time
10	67	Male	Development of LCOS in ICU stay	Yes	Yes – system	System error, early discharge from ICU
11	65	Male	Stroke, pre-operative normal sinus rhythm and postoperative atrial fibrillation-related cerebral infarct and emboli due to intracardiac thrombi	No	Yes – system	System error, lack of cardioversion on time
12	55	Male	Stroke	No	No	
13	57	Male	Stroke	No	No	
14	46	Male	Stroke	No	No	
15	54	Male	Stroke	No	No	
16	53	Female	Stroke	No	No	
17	59	Female	Stroke	No	No	
18	68	Male	Stroke	No	No	
19	58	Male	Mediastinitis	No	No	
20	58	Female	Mediastinitis	No	No	
21	55	Male	Pulmonary emboli	No	No	
22	54	Male	Respiratory failure, bullous lung disease	No	No	
23	54	Male	Ischaemic heart disease, poor distal run-off	Yes	No	
24	61	Male	Sudden cardiac death after discharge	Yes	No	Possible late tamponade?

CABG: coronary artery bypass grafting, MI: myocardial infarction, ICU: intensive care unit, LIMA: left internal mammarian artery, LCOS: low-cardiac output syndrome.

Among those 13 patients (54.2%) who were categorised as suffering unpreventable deaths, seven patients were diagnosed with a stroke, two had sepsis due to mediastinitis, one had pulmonary emboli (PE) without prominent deep-vein thrombosis, and another had respiratory insufficiency postoperatively. One patient had sudden cardiac arrest after discharge, and the last patient died due to ischaemic heart disease and possible poor distal run-off, causing a hypotensive cardiopulmonary failure in the ward that was not responsive to cardiopulmonary resuscitation. This patient was weaned off bypass with the help of intra-aortic balloon pump (IABP), but unfortunately died on the sixth day after the operation.

The patient who died of PE had been re-admitted to the intensive care unit (ICU) with sudden sharp chest pains and respiratory insufficiency six days after surgery. The sudden onset of symptoms and a chest X-ray suggested PE. The patient succumbed to respiratory failure 11 days after surgery. The other patient who died of respiratory failure had chronic obstructive pulmonary disease (COPD). After the operation, mechanical ventilatory support was discontinued on postoperative day four, however, hypoxaemia, possibly due to atelectasis and a ventilation–perfusion mismatch, required re-intubation. Death occurred due to respiratory failure on postoperative day six.

Eleven patients (45.8%) in the group of 24 deaths were found to have the cause of death categorised as preventable. Six were deemed technical errors and five system errors. Among the preventable deaths due to technical errors, all six patients suffered graft thrombosis. In five of the patients, graft thrombosis was encountered due to LIMA thrombosis. These patients developed low-cardiac output syndrome (LCOS) in the early postoperative period, requiring cardiopulmonary resuscitation and re-exploration, which showed dissection of the LIMA or haematoma at the LIMA wall. Although in two of these cases, the left anterior descending (LAD) coronary artery was bypassed with a saphenous vein graft for a second time, death was inevitable but secondary to peri-operative MI.

In the sixth patient who had graft thrombosis, ventricular fibrillation occurred on the third day after surgery and was the cause of death. Emergency cardiopulmonary bypass (CPB) was initiated after cardiopulmonary resuscitation. During surgical exploration, two thrombosed saphenous vein grafts were detected.

Five of the patients in the preventable death group were considered to have suffered a system error. The two patients who died of renal failure had excessive bleeding in the early postoperative period. Haemodialysis was initiated in both patients but they succumbed on the sixth and eighth days after surgery, respectively. One of the patients had atrial fibrillation (AF) but there was no attempt at cardioversion, possibly due to lack of communication between the consultant and the junior on-call doctor; the final cause of death was stroke.

Another patient, diagnosed with bullous lung disease, had subcutaneous emphysema and after an episode of sudden respiratory insufficiency, died in the ward on day 19 after surgery. A further patient with hypotension was transferred to the ward by a junior surgeon on postoperative day three, without considering that the clinical status of the patient required continuous inotropic support. This system error led to low-cardiac output syndrome and subsequent cardiac arrest. The patient failed to respond to cardiopulmonary resuscitation in the ward.

## Discussion

During open-heart surgery, patients with a high risk for cardiac surgery are extensively studied and there is substantial documentation of successful surgical outcomes in this group of patients.^8^,^9^ However, there is not enough data on preventable deaths in low-risk groups of patients. The investigation of low-risk groups with a EuroSCORE ≤ 2 requires a larger group of patients to demonstrate accurate mortality rates and it is often more difficult to predict the possible risk factors that influence outcomes of mortality in a small group.

In a study by Freed *et al.*,^10^ in a group of 4 294 patients with a logistic EuroSCORE ≤ 2, a total of 16 patients (0.37%) died. It has been claimed that over a third of the deaths studied were potentially preventable, suggesting that further improvement in outcomes is possible through modification of surgical technique or a change in the systematic delivery of cardiac surgical care and training.

In our study group, of a total of nine patients (37.5%) with peri-operative myocardial infarction and cardiac-related death, seven (77.8%) were considered to be preventable, and in five patients, the main problem was identified as LIMA harvesting. LIMA–LAD bypass is the mainstay of coronary surgery.^12^,^13^ The LIMA is used extensively as a bypass graft, with excellent patency rates.^13^,^14^ However, careful technical preparation is needed to prevent occlusion.

In other studies on isolated CABG, mainly CPB, cardioplegic solution, problems in myocardial protection, and the length of aortic cross-clamp were considered to be factors that may have increased cardiac-related mortality.^10^-^14^ Our study found that in five of the nine patients (55.6%) who suffered cardiac-related death, technical problems in LIMA harvesting was the most important cause of cardiac origin.

Two patients also died of renal failure as a result of excessive blood transfusions. Meticulous haemostasis and early exploration for postoperative bleeding may help to prevent excessive blood transfusion and therefore the development of renal insufficiency that requires dialysis, which is well known to increase mortality.^15^

In one patient, stroke was the reason for death, due to postoperative atrial fibrillation. However, the cause of death was secondary to a miscommunication between the junior surgeon on the ward and the senior surgeon, which caused a preventable system error. Early cardioversion is crucial and potentially lifesaving in the face of acute rhythm disturbances, which require immediate intervention. Late detection in the ward, miscommunication between the surgical team, and inability to transfer the patient to the ICU on time are all system-related errors that need to be identified and solutions discussed in order to prevent further fatalities.

In another patient who was still in the ward under medical supervision, underestimation of respiratory insufficiency due to pneumothorax was the cause of death. A further patient with clinical signs of deterioration of the haemodynamic status was transferred to the ward by a junior surgeon who underestimated the clinical status of the patient, leading to a system error and death. These system errors can be corrected by more established protocols and closer follow up of patients after surgery. Common to both the above cases, delay in the treatment of an identified and potentially reversible problem was recognised. The cause of this delay in taking appropriate action was the lack of or unclear communication between senior and junior surgeons.

There are several similar studies but they differed from our study in several ways, as the current study was designed to understand the details of mortality in low-risk cardiac surgery patients. In both the FIASCO study^10^ and the Stockholm experience,^11^ all cardiac surgical procedures were included, whereas our study dealt with only CABG surgery. Another difference from the FIASCO study was that patients were included who died within 30 days of surgery. Previous studies have only included in-hospital mortality, similar to the study by Janiec *et al.*^11^

The Stockholm experience has revealed excellent results (0.38% mortality), even in patient groups with a EuroSCORE ≤ 3. However, the incidence of preventable deaths was only 13% (2/15) of all deaths. In the FIASCO study, the incidence of mortality was similar to that of the Stockholm study (0.37%) in a group of low-risk patients with a logistic EuroSCORE ≤ 2. The incidence of preventable causes of death was 43%, markedly higher than that of the Stockholm experience.^10^,^11^

In our study, the mortality rate (0.93%) was higher than in both the above studies. Most importantly, the incidence of preventable death was found to have occurred in 11 patients (45.8%) in the group. Therefore we argue that reduction of technical surgical errors and system errors will improve the outcomes of this low-risk patient group. The main areas for improvement are seen as: (1) meticulous LIMA harvesting, (2) improvement in surgical technique for haemostasis during the surgical procedure, and (3) established protocols for patient care in the ICU and ward.

There were two limitations of the study; one was that the number of deaths in this group (24/2 570) still constitutes a relatively small number of patients from which to determine the real risk factors influencing mortality. A larger patient group or the pooling of patients through collaboration between hospitals will yield valuable data on this subject. The second major limitation of our study was the lack of an objective definition of preventable and non-preventable causes of death. In order to categorise a death as preventable or non-preventable, we had to rely on consensus between the authors and an expert external to the study, while searching for similar studies in the literature.^10^,^11^

## Conclusion

This study was conducted because there is insufficient data on the causes of preventable deaths in the low-risk group of patients undergoing isolated CABG. A structured analysis of the events preceding an unexpected fatality in patients with no or minimal risk factors should reveal potentially correctable issues. Furthermore, the correction of technical and system errors, such as LIMA harvesting and haemostasis during surgery, as well as the establishment of protocols for transferring patients from the ward to the intensive care unit will eventually lead to improvement in the quality of care and surgical outcomes.
